# Randomized controlled clinical trial on bleaching sensitivity and whitening efficacy of hydrogen peroxide versus combinations of hydrogen peroxide and ozone

**DOI:** 10.1038/s41598-018-20878-0

**Published:** 2018-02-05

**Authors:** Mahmoud K. AL-Omiri, Abdullah A. Al Nazeh, Andrej M. Kielbassa, Edward Lynch

**Affiliations:** 1 Department of Prosthodontics, School of Dentistry, University of Jordan, Queen Rania Street, Amman, 11942 Jordan; 2 Department of Prosthodontics, The City of London School of Dentistry, Brierley Price Prior (BPP) University, Canada Water, Lower Road, London SE16 2XB, United Kingdom; 3 Department of Paediatric Dentistry and Orthodontics, College of Dentistry, King Khalid University, King Fahad Street, Asir-Abha, 61471 Saudi Arabia; 4 Centre for Operative Dentistry, Periodontology, and Endodontology, University of Dental Medicine and Oral Health, Danube Private University (DPU), Steiner Landstraße 124, 3500 Krems, Austria; 5 Biomedical and Clinical Research, School of Dental Medicine, University of Nevada (UNLV), 1001 Shadow Lane, Las Vegas, Nevada 89106-4124 United States of America

## Abstract

The clinical efficacy regarding bleaching sensitivity and tooth shade lightening using a standard hydrogen peroxide (H_2_O_2_) bleaching gel was compared with the additional use of ozone either before or after application of H_2_O_2_. Using computer-generated tables, 45 participants were randomly allocated into three groups (n = 15 each) in this investigator-driven, single-centre trial. In Group 1, upper anterior teeth were bleached using ozone (produced via a healOzone X4 device) for 60 seconds, then 38% H_2_O_2_ for 20 minutes; in Group 2, 38% H_2_O_2_ application (20 min) was followed by ozone (60 s); air produced by the healOzone machine (60 s) followed by 38% H_2_O_2_ (20 min) was used in Group 3 (control). Bleaching sensitivity was evaluated via visual analogue scales, and a treatment-blinded reader objectively recorded tooth shades using a colorimeter before and 24 hours after bleaching (at α = 0.05). The H_2_O_2_/ozone combination did not result in pain sensations, while both ozone/H_2_O_2_ and H_2_O_2_ alone increased bleaching sensitivity (p < 0.001). Teeth achieved lighter shades (higher L*/lower b* values) after bleaching in all groups (p < 0.001), while Ozone boosted lighter tooth shades, irrespective of its use before or after H_2_O_2_ (p < 0.05). Due to the complimentary effects, applying ozone after H_2_O_2_ seems preferable for bleaching.

## Introduction

Ozone (O_3_) is a strong oxidizing agent able to destroy bacteria, viruses, fungi, yeasts and protozoa as well as odours, and has been utilized for a long time in medicine and dentistry^1–5^. Beneath various other dental purposes, ozone has been applied for lightening of teeth in recent years^6–15^, but results from the available literature have been contradictory, at least to some extent. Some authors reported that using ozone did not potentiate the bleaching ability of 8% carbamide peroxide, and even reduced the bleaching efficacy if applied before 8% carbamide peroxide^15^, while others concluded that hydrogen peroxide (H_2_O_2_) had superior bleaching capacities if compared to ozone alone^6^, and that ozone did not improve the bleaching effectiveness of 35% hydrogen peroxide^12^.

In contrast, ozone alone has been reported to have similar bleaching capacities like some commercially available, highly concentrated carbamide peroxides (45%)^13^ or hydrogen peroxide (37.5%)^11^ bleaching agents. In addition, ozone was found to improve the shades of tetracycline stained rat teeth^14^. Moreover, 30% carbamide peroxide has been shown to have inferior bleaching outcomes if compared to ozonated gel when used to bleach stained resin composite discs^16^. Finally, our working group recently has revealed that 38% H_2_O_2_ offered superior bleaching results when used together with ozone^7,9,10^, and we found that ozone gas had similar bleaching results to 38% H_2_O_2_^8^.

Notwithstanding, the available literature on bleaching action of ozone suffers from some limitations such as investigating low ozone or peroxide dosage and concentrations^12,15^, small study sample sizes^12,15,16^, using older models of ozone-generating machines^13,15,17^, inability to measure ozone concentrations produced by ozone machines^12,14,15^, following study designs not accurately imitating clinical conditions^13,15^, studying bleaching results on artificial extrinsic tea stains (but not the colour of dental structures)^12,15^, measuring hue component of tooth shade only^14^, or subjectively utilizing visual shade guides to measure tooth shades (without objective standardization)^13,14^. Therefore, concluding recommendations regarding bleaching efficacy are hardly educable from the current literature.

In medicine, locally applied ozone has been shown to alleviate painful conditions^18,19^, and to reduce inflammatory responses^20,21^. So far, however, no prior clinical trial has explored the results of utilizing ozone before H_2_O_2_ application in comparison to using ozone after H_2_O_2_ application for dental bleaching, and no information is retrievable with regards to prevailing pain sensations due to tooth whitening procedures, a phenomenon called bleaching sensitivity^22^. This inspired the current study to better understand the bleaching effects of ozone on discoloured teeth before or after the use of H_2_O_2_.

Hence, the aim of this investigation was to study tooth sensitivity and the clinical efficacy of tooth bleaching using both ozone/H_2_O_2_ gel and H_2_O_2_ gel/ozone treatment sequences, and to compare these with conventional bleaching using H_2_O_2_ gel only. The null hypotheses (H_0_) for this study were that applying ozone to teeth surfaces would not result in different tooth sensitivity or bleaching outcome if used before or after 38% H_2_O_2_, and that bleaching with ozone and H_2_O_2_ would produce similar effects compared to bleaching with H_2_O_2_ alone. These null hypotheses were tested against the alternative hypotheses of a difference (H_A_).

## Methods

This investigation was organised in full ethical accordance with the Helsinki Declaration of 1964 (as revised and amended in its ninth version in 2013)^23^. Approval of the study protocol by the Deanship of Research, University of Jordan, Amman, Jordan (ethical vote number ARC-5-2015) was obtained, and all participants of this three-arm clinical trial gave their written informed consent for participation and the use of their respective data for research purposes. Blindness of the evaluator regarding the respective treatments in the assigned groups of the patients was assured. With the present report, we adhered to the CONSORT statement on reporting randomized trials^24^.

### Sample size calculation

Sensitivity of teeth (24 h after beaching) was defined as the primary endpoint of the current study. Using the pooled variance based on our previous study^9^, we computed an effect size of 0.544 (G*Power: Statistical Power Analyses, version 3.1.9.3; Heinrich-Heine University)^25^ for an *a-priori* power analysis. A sample size of 12 per group was calculated as a function of the required power level (1 – β; 0.8), the pre-specified significance level α (0.05), and the population effect size to be detected with probability 1 − β^25^. In total, 15 participants were finally selected for each group to compensate for any unexpected (but sometimes inevitable) dropouts. The humane endpoint was defined as the point at which pain induced by the bleaching procedure was not bearable anymore^26^; in these cases, terminating the possibly painful procedure was scheduled.

### Recruitment of patients

**Figure 1:**
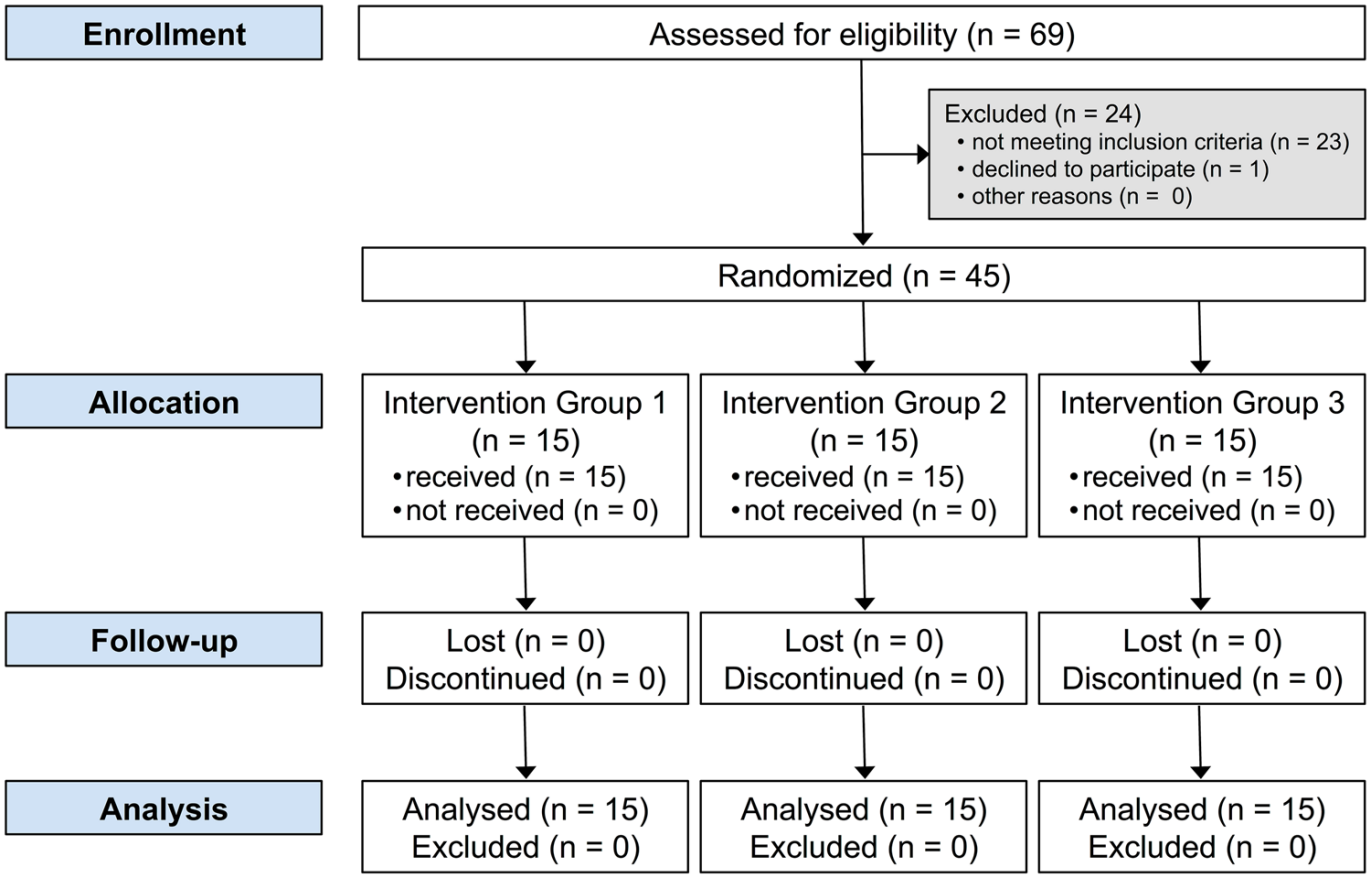
Graphic depiction of flow of participants through each stage of randomized trial. Group 1 = Teeth bleached with ozone then 38% hydrogen peroxide; Group 2 = Teeth bleached with 38% hydrogen peroxide then ozone; Group 3 (control) = Teeth bleached with 38% hydrogen peroxide only.

A total of 69 patients interested in this study were examined (Fig. 1). Forty-five participants (24 females and 21 males) were finally recruited into this study. All participants were regular patients visiting the dental clinics at the University of Jordan, and searching for bleaching treatment.

Each participant received a detailed explanation of the study and the involved procedures (along with the potential side effects)^27^ and was requested to provide written informed consent before being recruited into the study. Participants were included in this investigation if they had all their upper anterior teeth (from right canine to left canine) present and sound, if their teeth had never been bleached before, and if they had no previous prosthetic, endodontic or restorative treatment for their upper anterior teeth. Vitapan classical shades (Vita Zahnfabrik, Bad Säckingen, Germany) should be A3 or darker^28^, and lighter shades were not included. Participants who had missing upper anterior teeth or had their upper anterior teeth affected by carious lesions, periodontitis, recession, extensive tooth surface loss, or any other complicating medical history as well as pregnant/lactating women were excluded from the present study.

### Randomisation

Subsequently, the participants were randomly allocated into three groups (n = 15 for each group), and the teeth were bleached as described below. A simple randomization process using computer-generated numbers was followed to distribute participants to the three treatment groups. To avoid any sex-based bias^29^, stratification according to gender was ensured.

### Examination of patients

A comprehensive clinical examination was carried out on a dental chair equipped with a light unit (Diamond LED Dental Light; Daray Lighting, Derbyshire, England, UK). The upper anterior teeth received a prophylaxis using pumice and water, and were dried before being examined. An explorer probe (0700-9, anatomical handle single ended; ASA Dental, Bozzano, Italy) and a dental oral mirror (15/16 inch; Hahnenkratt, Königsbach-Stein, Germany) were used throughout all intra-oral examinations. If needed, the teeth were scaled and polished before commencing the investigation.

Then, tooth sensitivity was evaluated via a visual analogue scale (VAS) ranging from 0 (‘no tooth sensitivity at all’) to 10 (‘pain as bad as imaginable’). Following previous recommendations^9,30^, the tooth shades (from upper right canine to upper left canine) were evaluated objectively using a chroma meter (CR-400; Minolta, Osaka, Japan)^28^ from a standard distance (7 cm away from the measured tooth surface) while the participant was sitting upright in the dental unit. The colour-measuring device was placed on a movable metal tray connected to the vertical column of the dental chair light unit. A 7 cm long plastic rod was inserted between the centre of the colorimeter orifice and the centre of the assessed tooth surface to ensure that the colorimeter was repeatedly fixed at 7 cm from the tooth surface before starting the shade measurement. Standardization of lighting conditions was ensured by carrying out all shade measurements under natural daylight with the clinic room lights and the dental unit light on (but turned away from the study participant). All teeth to be examined were moistened with water from a 3-in-1 syringe. To standardize the measuring procedure, shade measurements were carried out following the same order for each participant, from the right canine to the left one. The colorimeter (CR-400; Minolta) recorded Vitapan classical shades (Vita Zahnfabrik) using descending values from light to dark as well as L*a*b* shade values by measuring the intensity of reflected visible light for red, green, yellow and blue wave lengths using the L*a*b* coordinates of colour arrangement in the CIELab colour scheme^9,10^.

### Bleaching intervention

With all patients, a light-curing dental dam covering and protecting the gingival tissues (BMS white BM; BMS Dental, Capannoli, Italy) was used. Participants’ teeth in Group 1 were bleached by application of ozone for 60 seconds on the labial surface of each tooth; a 2,350 ppm ozone concentration at a 615 cc per minute flow rate was supplied by a well-known ozone producing machine (healOzone X4; Curozone, Wiesbaden, Germany)^7,9,17^. The ppm of ozone supplied was verified via an ozone detection device, and the ozone flow rate was verified by a flow meter directly before the start of the experiment. Ozone gas was distributed to the tooth surfaces through disposable silicone cups provided by the manufacturer and assured a perfect seal to prevent any ozone leakage. The healOzone X4 machine only supplies ozone once the cup provides an absolute seal; this element allows the machine to be safely employed to humans^17,31^. Subsequently, application of 38% H_2_O_2_ gel (BMS white 38%; BMS Dental) followed for 20 minutes. Then, the H_2_O_2_ gel was removed and the teeth were sprayed for 10 seconds with water from a 3-in-1 syringe.

In comparison to Group 1, participants’ teeth in Group 2 were first bleached by application of 38% H_2_O_2_ (BMS white 38%; BMS Dental) gel for 20 minutes, and then sprayed for 10 seconds with water from a 3-in-1 syringe. Subsequently, ozone was applied on each tooth surface for 60 seconds.

In contrast, participants’ teeth in Group 3 were exposed to 60 seconds of air only (no ozone) provided by the ozone machine (which was specifically modified, and was achieved by using a switch on the back). A 38% H_2_O_2_ gel (BMS white 38%; BMS Dental) was applied on the teeth for 20 minutes, and then washed for 10 seconds with water from a 3-in-1 syringe.

### Follow-up

With the initial rebound of colour effect in mind, all participants were dismissed and requested to return 24 hours later to permit rehydration of tooth surfaces before shade and tooth sensitivity assessment. Tooth shades were then recorded using the already described chroma meter (CR-400; Minolta), and tooth sensitivities were evaluated using the VAS scale from 0 to 10 as explained above.

### Statistical analysis

Assigning patients to interventions and tooth bleaching was accomplished by one investigator (M.K.A.-O.), while all shade measurements and tooth sensitivity assessments were carried out by another investigator (A.A.A.N.), who was blinded to the respective bleaching technique. Intra-examiner reliability was evaluated by recording 18 duplicate shade measurements by the same investigator and Kappa was considered adequate (κ** = **0.91; almost perfect conformity), thus proving high intra-examiner agreement of the standardized assessment methods.

The SPSS computer software (Statistical Package for the Social Sciences, v19.0; IBM, Armonk, NY, USA) was utilized to carry out data analysis for the current study. Paired samples *t*-test was executed to compare shade values before and after bleaching within each group. Analysis of variance (ANOVA) was performed to compare shade values between groups. *Post-hoc* test was carried out for additional comparisons of shade values between groups at baseline and after bleaching. Statistically significant levels were set at p < 0.05, with a 95% confidence interval. A *post-hoc* power calculation analysis based on the sensitivity means and standard deviations was conducted to compute the actual power level (G*Power, version 3.1.9.3; Heinrich-Heine University, Düsseldorf, Germany)^25^.

## Results

This investigation took place at the Department of Prosthodontics, University of Jordan (September 2016 till March 2017). From the initially 69 screened patients, 24 were excluded; reasons for exclusion of participants are given in Fig. 1. An overall of 270 upper anterior teeth in 45 participants were finally included and investigated in the current study. Participants’ age ranged between 19 and 33 years (mean ± SD = 25 ± 4 years). In each group, 8 participants were female.

### Bleaching sensitivity

Table 1 demonstrates the means and standard deviations of the levels of tooth sensitivity and recorded Vita and L*a*b* shade values among the study groups at study baseline and following the respective bleaching sequences. None of the teeth in this clinical experiment was affected by sensitivity at baseline. However, Groups 1 (first ozone, then H_2_O_2_ bleaching) and 3 (H_2_O_2_ only controls) revealed some sensitivity after bleaching (p < 0.001) (Table 2). On the other hand, teeth in Group 2 (first H_2_O_2_, then ozone bleaching) did not show any bleaching sensitivity at all. Regarding bleaching sensitivity, the *post-hoc* power analysis resulted in a level considered adequate (>99.7%) to detect a clinically relevant difference between the outcomes of the three study arms.

**Table 1:** Distribution of VAS tooth sensitivity scores and L*a*b* shade values [means ± standard deviations (SD)] before and after bleaching of teeth.

	Group 1		Group 2		Group 3	
	Baseline	After bleaching	Baseline	After bleaching	Baseline	After bleaching
Sensitivity	0.00 (0.00)	3.20 (2.21)	0.00 (0.00)	0.00 (0.00)	0.00 (0.00)	1.60 (1.78)
Vita shades	10.52 (1.40)	14.80 (2.35)	10.41 (1.25)	15.04 (1.47)	10.45 (1.30)	13.18 (1.54)
L* value (SD)	83.01 (5.98)	86.43 (3.71)	84.32 (6.03)	87.40 (5.73)	84.37 (6.02)	85.82 (5.42)
a* value (SD)	−2.39 (2.58)	−2.70 (1.88)	−3.32 (2.75)	−3.97 (2.81)	−3.31 (2.75)	−3.85 (2.49)
b* value (SD)	16.70 (4.61)	11.97 (3.88)	14.77 (6.78)	10.50 (5.06)	14.54 (6.75)	11.88 (6.53)

Group 1 = Teeth bleached with ozone then 38% hydrogen peroxide (n = 90 tooth surfaces in 15 participants).

Group 2 = Teeth bleached with 38% hydrogen peroxide then ozone (n = 90 tooth surfaces in 15 participants).

Group 3 (control) = Teeth bleached with 38% hydrogen peroxide only (n = 90 tooth surfaces in 15 participants).

**Table 2:** Paired samples *t*-test for within group variations in tooth sensitivity and shade values of teeth at baseline and following bleaching.

Group	Sensitivity/Vita shade pairs	Paired Differences			t	df	Sig. (2-tailed)
		Std. Error Mean	95% Confidence Interval of the Difference				
			Lower	Upper			
1	Sensitivity baseline – Sensitivity final	0.23288	−3.66272	−2.73728	−13.741	89	0.000
	Vita baseline – Vita final	0.28222	−5.56077	−4.43923	−17.716	89	0.000
	L* baseline – L* final	0.43369	−4.28218	−2.55871	−7.887	89	0.000
	a* baseline – a* final	0.20215	−0.09622	0.70711	1.511	89	0.134
	b* baseline – b* final	0.35296	4.02424	5.42688	13.388	89	0.000
2	Sensitivity baseline – Sensitivity final	0.0000	—	—	—	—	—^$^
	Vita baseline – Vita final	0.31586	−5.25918	−4.00273	−14.662	89	0.000
	L* baseline – L* final	0.59534	−4.26435	−1.89612	−5.174	89	0.000
	a* baseline – a* final	0.29322	0.06822	1.23464	2.222	89	0.029
	b* baseline – b* final	0.58641	3.09615	5.42885	7.269	89	0.000
3	Sensitivity baseline – Sensitivity final	0.19316	−1.98412	−1.21588	−8.283	89	0.000
	Vita baseline – Vita final	0.26192	−4.27379	−3.23209	−14.329	89	0.000
	L* baseline – L* final	0.39920	−2.23797	−0.65027	−3.618	89	0.001
	a* baseline – a* final	0.24353	0.06112	1.02970	2.240	89	0.028
	b* baseline – b* final	0.55738	1.55911	3.77595	4.786	89	0.000

Group 1 = Teeth bleached with ozone then 38% hydrogen peroxide (n = 90 tooth surfaces in 15 participants).

Group 2 = Teeth bleached with 38% hydrogen peroxide then ozone (n = 90 tooth surfaces in 15 participants).

Group 3 (control) = Teeth bleached with 38% hydrogen peroxide only (n = 90 tooth surfaces in 15 participants).

t = t-test statistics; df = degree of freedom; Sig = Significance (P value).

^**$**^Paired difference could not be computed for tooth sensitivity in Group 2 because the standard error of difference equals zero.

### Bleaching outcome

In addition, all bleaching techniques caused changes in L*a*b* shade values, and the investigated teeth acquired lighter shades (p < 0.001) (Table 2). Also, L* shade values were enhanced (leading to lighter shades) following bleaching with ozone then H_2_O_2_ in Group 1 (p < 0.001), following bleaching with H_2_O_2_ then ozone in Group 2 (p < 0.001), and following bleaching with H_2_O_2_ alone in Group 3 (p = 0.001) (Table 2). Moreover, b* shade values were reduced (leading to lighter shades) following bleaching with ozone then H_2_O_2_ in Group 1 (p < 0.001), following bleaching with H_2_O_2_ then ozone in Group 2 (p < 0.001), and following bleaching with H_2_O_2_ alone in Group 3 (p < 0.001) (Table 2). In contrast, a* shade values did not significantly change following bleaching with ozone then H_2_O_2_ in Group 1 (0 = 0.134). Notwithstanding, a* shade values were significantly decreased following bleaching with H_2_O_2_ then ozone in Group 2 (p = 0.029), and following bleaching with H_2_O_2_ alone in Group 3 (p = 0.028) (Table 2).

Comparisons between groups using ANOVA demonstrated that baseline tooth sensitivity, Vita shades, and L*a*b* shade values were comparable between groups (p > 0.05) (Table 3). In contrast, tooth sensitivity following bleaching was significantly different between groups (p < 0.001). In addition, final Vita shades acquired after bleaching were significantly different between groups (p = 0.03) (Table 3). On the other hand, final L* and b* shade values were not significantly different between groups (p > 0.05) (Table 3), whereas a* values were significantly different between groups (p = 0.001) (Table 3).

**Table 3:** Analysis of variance (ANOVA) of shade values between groups at baseline and after bleaching (n = 90 tooth surfaces in 15 participants for each group).

Sensitivity/Shade value		Sum of Squares	df	Mean Square	F	Sig. (*P* value)
Baseline sensitivity	Between groups	0.000	2	0.000	—	—
	Within groups	0.000	267	0.000		
	Total	0.000	269			
Final Sensitivity	Between groups	445.084	2	222.542	81.294	0.000
	Within groups	700.800	267	2.738		
	Total	1145.884	269			
Baseline Vita	Between groups	22.169	2	11.084	0.799	0.451
	Within groups	3553.391	256	13.880		
	Total	3575.560	258			
Final Vita	Between groups	33.458	2	16.729	3.540	0.030
	Within groups	1209.646	256	4.725		
	Total	1243.104	258			
Baseline L*	Between groups	106.072	2	53.036	1.470	0.232
	Within groups	9238.318	256	36.087		
	Total	9344.390	258			
Baseline a*	Between groups	49.759	2	24.879	1.054	0.340
	Within groups	1859.914	256	7.265		
	Total	1909.673	258			
Baseline b*	Between groups	246.748	2	123.374	1.013	0.380
	Within groups	9533.279	256	37.239		
	Total	9780.027	258			
Final L*	Between groups	108.312	2	54.156	2.159	0.118
	Within groups	6420.684	256	25.081		
	Total	6528.995	258			
Final a*	Between groups	87.000	2	43.500	7.476	0.001
	Within groups	1489.660	256	5.819		
	Total	1576.659	258			
Final b*	Between groups	114.999	2	57.499	2.088	0.126
	Within groups	7050.648	256	27.542		
	Total	7165.647	258			

df = Degree of Freedom; Sig. = Significance; F = F statistics.

Additional comparisons between study groups by means of *post-hoc* statistics (Table 4) showed that baseline tooth sensitivity, Vita shades and L*a*b* shade values were not significantly different between any two groups (p > 0.05) (Table 4). Following bleaching, teeth in Group 2 revealed significantly less bleaching sensitivity than teeth in Groups 1 and 3 (p < 0.001), while teeth in Group 3 had less sensitivity than teeth in Group 1 (p < 0.001). Consequently, application of ozone after H_2_O_2_ was associated with less sensitivity following bleaching.

**Table 4:** *Post-hoc* test of tooth sensitivity and shade value variations between groups before and after bleaching.

Dependent Variable	(I) Group	(J) Group	(I-J) Mean Difference	Std. Error	Sig.	95% CI	
						Lower Bound	Upper Bound
Sensitivity after bleaching^$^	1	2	3.20000*	0.25101	0.000	2.6083	3.7917
		3	1.60000*	0.25024	0.000	1.0101	2.1899
	2	1	−3.20000*	0.25101	0.000	−3.7917	−2.6083
		3	−1.60000*	0.25455	0.000	−2.2001	−0.9999
	3	1	−1.60000*	0.25024	0.000	−2.1899	−1.0101
		2	1.60000*	0.25455	0.000	0.9999	2.2001
Baseline Vita	1	2	−0.60476	0.56522	0.534	−1.9372	0.7277
		3	−0.62353	0.56349	0.511	−1.9519	0.7049
	2	1	0.60476	0.56522	0.534	−0.7277	1.9372
		3	−0.01877	0.57319	0.999	−1.3700	1.3325
	3	1	0.62353	0.56349	0.511	−0.7049	1.9519
		2	0.01877	0.57319	0.999	−1.3325	1.3700
Final Vita	1	2	−0.23571	0.32978	0.755	−1.0131	0.5417
		3	0.62353	0.32877	0.142	−0.1515	1.3986
	2	1	0.23571	0.32978	0.755	−0.5417	1.0131
		3	0.85924*	0.33443	0.029	0.0709	1.6476
	3	1	−0.62353	0.32877	0.142	−1.3986	0.1515
		2	−0.85924*	0.33443	0.029	−1.6476	−0.0709
Baseline L*	1	2	−1.31860	0.91136	0.319	−3.4671	0.8299
		3	−1.36773	0.90858	0.290	−3.5097	0.7742
	2	1	1.31860	0.91136	0.319	−0.8299	3.4671
		3	−0.04913	0.92421	0.998	−2.2279	2.1296
	3	1	1.36773	0.90858	0.290	−0.7742	3.5097
		2	0.04913	0.92421	0.998	−2.1296	2.2279
Baseline a*	1	2	0.92588	0.40892	0.063	−0.0381	1.8899
		3	0.91508	0.40768	0.066	−0.0460	1.8761
	2	1	−0.92588	0.40892	0.063	−1.8899	0.0381
		3	−0.01080	0.41469	1.000	−0.9884	0.9668
	3	1	−0.91508	0.40768	0.066	−1.8761	0.0460
		2	0.01080	0.41469	1.000	−0.9668	0.9884
Baseline b*	1	2	1.92951	0.92580	0.095	−0.2530	4.1120
		3	2.15146	0.92297	0.053	−0.0244	4.3273
	2	1	−1.92951	0.92580	0.095	−4.1120	0.2530
		3	0.22195	0.93885	0.970	−1.9913	2.4352
	3	1	−2.15146	0.92297	0.053	−4.3273	0.0244
		2	−0.22195	0.93885	0.970	−2.4352	1.9913
Final L*	1	2	−0.97840	0.75977	0.199	−2.4746	0.5178
		3	0.60859	0.75746	0.422	−0.8830	2.1002
	2	1	0.97840	0.75977	0.199	−0.5178	2.4746
		3	1.58699*	0.77049	0.040	0.0697	3.1043
	3	1	−0.60859	0.75746	0.422	−2.1002	0.8830
		2	−1.58699*	0.77049	0.040	−3.1043	−0.0697
Final a*	1	2	1.27187*	0.36596	0.002	0.4091	2.1346
		3	1.15505*	0.36485	0.005	0.2949	2.0151
	2	1	−1.27187*	0.36596	0.002	−2.1346	−0.4091
		3	−0.11682	0.37112	0.947	−0.9917	0.7581
	3	1	−1.15505*	0.36485	0.005	−2.0151	−0.2949
		2	0.11682	0.37112	0.947	−0.7581	0.9917
Final b*	1	2	1.46645	0.79617	0.158	−0.4105	3.3434
		3	0.09343	0.79375	0.992	−1.7778	1.9646
	2	1	−1.46645	0.79617	0.158	−3.3434	0.4105
		3	−1.37302	0.80740	0.207	−3.2764	0.5304
	3	1	−0.09343	0.79375	0.992	−1.9646	1.7778
		2	1.37302	0.80740	0.207	−0.5304	3.2764

Group 1 = Teeth bleached with ozone then 38% hydrogen peroxide (n = 90 tooth surfaces in 15 participants).

Group 2 = Teeth bleached with 38% hydrogen peroxide then ozone (n = 90 tooth surfaces in 15 participants).

Group 3 (control) = Teeth bleached with 38% hydrogen peroxide only (n = 90 tooth surfaces in 15 participants).

Sig. = Significance (P value); *Significant difference; CI = Confidence Intervals.

^**$**^Differences cannot be computed for baseline tooth sensitivity because the mean difference and the standard error of difference equals zero.

Finally, Group 1 was not significantly different from Groups 2 and 3 regarding final Vita Classic shades and final L* and b* shade values (p > 0.05) (Table 4). In contrast, Group 1 revealed higher final a* shade values (darker shades) if compared to Groups 2 (p = 0.002) and 3 (p = 0.005). Also, Group 2 had lighter final Vita Classic shades (p = 0.029) and higher final L* shade values (lighter shades) (p = 0.04) than Group 3. Therefore, bleaching with H_2_O_2_ then ozone produced lighter shades than bleaching with H_2_O_2_ alone. Groups 2 and 3 were not significantly different regarding final a* and b* shade values (p > 0.05) (Table 4).

### Harms and unintended side effects

Apart from the bleaching sensitivities observed in Groups 1 and 3 (this was regarded as a predictable and common side effect), we did not observe any harms or unintended effects, neither with one of the used materials nor in any of the study groups in the present study. None of the observed bleaching sensitivities was considered unbearable.

## Discussion

The current investigation showed that application of ozone after H_2_O_2_ was associated with no tooth sensitivity at all, and this was in line with a recent study^9^. Bleaching via 38% H_2_O_2_ followed by ozone resulted in effects comparable to bleaching with ozone followed by 38% H_2_O_2_. In addition, bleaching with ozone and H_2_O_2_ (in any application sequence) proved to be superior compared to H_2_O_2_ alone. Consequently, the null hypothesis of this study (speculating that no variation in efficacy would be observed between the three bleaching procedures) was rejected.

In the present study, shade evaluation was standardized through recording the shade from a fixed distance around mid-day within the same clinical settings for all participants, and the results were adequately reproducible. The healOzone appliance was used to provide ozone (or air only in the control group) since it has been shown to be safe as its ozone releasing system can be effectively sealed before the appliance supplies ozone^17,31^.

Regarding the baseline colours, most included teeth were of dark A shades, and most patients showed more than one shade with their anterior teeth (even after a thorough prophylaxis). In the present trial, we dealt with the original colours as numbers according to the Vita shade arrangement from lighter to darker shades (From B1 to C4) and numbers from 16 to 1 were given to the shades (B1 = 16, A1 = 15 to C4 = 1), according to their sequence in the Vita shade arrangement^28^. The analysis was done accordingly after assessing how many degrees the respective tooth had advanced on the Vita shade arrangement following bleaching; this was in accordance with previous investigations using computer-aided shade evaluations^28,32^.

Bleaching procedures were performed within clinical settings. Whitening by means of hydrogen peroxide was used as a control; this was not accompanied by another control subgroup (*i*. *e*. air after bleaching), since the latter was not considered to comply with clinical practice. The whitening gel used to treat the dental dyschromia (BMS White 38%; BMS Dental) contained 38% hydrogen peroxide, and thus was comparable to other in-office bleaching gels marketed worldwide. It is known that H_2_O_2_ can cause enamel etching due to release of protons (H^+^), thus opening tiny pores^26^.

From the present outcome it seems clear, that ozone and H_2_O_2_ successfully acted in concert to boost tooth shades. Ozone is a provider of superoxide (O˙), and could contribute additional hydroxyl radicals (OH˙) when combined with peroxides; this would suggest more effective bleaching capacities. Besides, ozone is classified as one of the most powerful oxidants (after fluorine and persulfate)^4^. Moreover, the synergistic dental bleaching actions of combined peroxides and ozone (a process called peroxonation) would seem in accordance with superior oxidation actions reported in areas other than dentistry^33,34^. Such advanced oxidative processes have been reported to promote oxidative degradation of endotoxins (induced by *in situ* generation of a more powerful oxidizing agent, such as hydroxyl radicals)^33,35^, thus decreasing the induction of cell signalling proteins involved in inflammation (*i*. *e*. tumour necrosis factors α), and reducing the inflammatory activities^1,20,35^.

Pain perception was measured by means of visual analogue scales (VAS). This valuable tool is generally accepted and has been widely used to evaluate pain sensations (or particular characteristics or attitudes) believed to represent a continuum of subjective data not assessable by objective measurements. In the present study, VAS pain scores as documented by the participants were significantly increased in both the ozone/H_2_O_2_ and the H_2_O_2_ alone groups. Regarding these observations, the *post-hoc* power analysis indicated that the present investigation had adequate power to meet the statistical requirement of a power level of at least 0.8.

Bleaching sensitivity is a well-known side effect of tooth whitening; however, this adverse reaction has not been fully understood up to now^22^. It is known that bleaching with high-concentrated hydrogen peroxides results in an increased expression of inflammatory mediators such as Substance P^36^, which in turn interacts with a great variety of cells, thus inducing the release of inflammatory mediators such as prostaglandins and cyclooxygenases^37^, which both have a recognized role in triggering nociceptive impulses for the perception of pain. Subsequently, both the concomitant increase in vascular permeability and the tissue pressure rise will result in pain, and this local inflammatory response of the dental pulp may be intense^38,39^.

In contrast, the findings of the current study revealed that bleaching sensitivity was not observed with the participants of the H_2_O_2_/ozone group. This observation might be attributed to the documented analgesic properties of ozone^18,19,40^. Topically applied ozone has been shown to exert ameliorative effects on lumbar disc herniation patients^21^, and low-concentrated (non-toxic) ozone concentrations have revealed potent anti-oxidant and anti-inflammatory effects on oxidative stress-induced tissue injuries^41^. Interestingly enough, exposure of human tracheal epithelial cells to ozone obviously results in a prolonged decrease in prostaglandin production^42^ and inactivates cyclooxygenase^43^, thus suggesting that the inflammatory pathways will be suppressed by ozone^1^.

Moreover, concentrations of vitality protector enzymes such as superoxide dismutase (an enzyme catalysing the conversion of the superoxide radical [O_2_^−^] into oxygen or hydrogen peroxide) have been reported to be low in healthy dental pulp tissue; with the proceeding of inflammatory responses, the pulp tissues showed a considerable adaptation to this situation^44^. Consequently, to find a large increase in catalase activity in inflamed pulp tissues would not seem surprising^45,46^. With a controlled application, ozone increases the activity of anti-oxidant enzymes (including catalase, glutathione peroxidase and superoxide dismutase), thus preparing the host to face pathophysiological and damaging conditions mediated by reactive hydrogen peroxide^4,41^. At present, these considerations undoubtedly are translational in nature, but confirming the anti-oxidative, anti-inflammatory and analgesic effects of ozone^20^ for dental pulp tissues would constitute a novel and momentous approach to combat bleaching sensitivity, and clearly merits further research.

Other possible explanations for the ozone-based reduced sensitivity have been presented in the available literature; these include decrease of number and diameter of open dentinal tubules^47,48^ and collagen degradation^38,49^, with potentially reduced sensitivities by mechanical blocking of the dentinal tubules. Moreover, some remineralisation of tooth surfaces in teeth bleached with H_2_O_2_/ozone might contribute to decreased pain perception, too. However, the aspects provided above are considered to take some time, and, therefore, would seem speculative at present.

In contrast, our present findings showed that using ozone before H_2_O_2_ was associated with the highest levels of sensitivity following bleaching. This effect might be in accordance with the synergistic function of ozone and peroxides for bleaching and handling of water pollutants or industrial wastes including in the textile industry; the latter procedure has been recognized as an advanced oxidative process^33,35^. A quick and potent oxidative consumption of coloured substances incorporated into enamel and/or dentin might have facilitated deeper penetration of hydrogen peroxide. It would seem conceivable that residual ozone remaining on the tooth surface has resulted in more advanced oxidative processes which in turn may have led to higher amounts of more free radicals reacting with the pulpal complex in a shorter time.

The secondary endpoint with respect to efficacy was the whitening effect after 24 hours (including the initial rebound after water sorption), and this set-up was conforming with a previous study^9^. It should be emphasised that secondary endpoints usually are lacking the same statistical authority if compared to the primary endpoint. Thus, positive effects with regard to secondary endpoints frequently are due to chance, should be interpreted with caution, and require α level correction for multiplicity; however, secondary endpoints would seem suitable to construe the primary result of a trial, and to demonstrate additional effects. Notwithstanding, it may be argued for the present outcome that efficacy with regard to bleaching outcome is strongly interlinked with pain perception; in other words, using hydrogen peroxide for in-office bleaching is a well-established clinical procedure commonly leading to bleaching sensitivity^22,26^, and any treatment option should strive for painless whitening. Hence, both endpoints (termed co‐primary endpoints) should achieve statistical significance to be considered clinically efficacious, and there is broad agreement that no multiplicity correction of the type I error is required in such situations^50^.

The outcomes of the current investigation revealed that lighter tooth shades (>4 Vita shades) were obtained following bleaching with both H_2_O_2_ and O_3_, irrespective of using ozone before or following hydrogen peroxide, and the teeth obtained significantly lighter shades in contrast to teeth bleached using H_2_O_2_ alone. This could be due to an additional and rapid production of free radicals (due to the ozone application) showing potent bleaching capacities and being capable of influencing tooth shades. Furthermore, this concurs with the results of previous studies concluding that ozone enhanced the shades of tetracycline stained rats’ incisor teeth^14^, and revealing bleaching outcomes comparable to high carbamide peroxide concentrations^13^. While a recent paper has elaborated that ozone (if used alone) does not outmatch the bleaching capacity of hydrogen peroxide^6^, the present outcomes harmonize with our previous investigations^7–10^, thus deducing that ozone boosted H_2_O_2_ dental bleaching. It would seem probable that some residual H_2_O_2_ or O_3_ may have remained in the porous system of the teeth prior to the following application of ozone or hydrogen peroxide, respectively, thus leading to advanced oxidative processes.

Notwithstanding, the outcomes of the current research contrast with another study having shown that 8% carbamide peroxide bleaching capacities would not be enhanced by ozone application and that using ozone before application of 8% carbamide peroxide would result in inferior bleaching outcomes^15^. This difference could be due to variations in sample size and study settings (as the respective study employed another ozone-producing device, supplying lower concentrations) which used ozone for 40 seconds, bleached external tea stains instead of internal tooth colour, and tested 8% carbamide peroxide. The latter is known to need a longer duration to effectively finish the bleaching process because of its low concentration providing 12 times less peroxides than the peroxide applied in the present study, and due to its mode of action first requiring a dissociation process to H_2_O_2_ and urea. Additionally, neither the supplied ozone concentration nor its flow rate had been reported^15^, thus not allowing for any further comparisons. Moreover, the findings of the present study disagree with the results of a previous investigation that has not uncovered any synergistic actions for ozone on H_2_O_2_ bleaching^12^. Again, this variation could be explained with teeth stained extrinsically by black tea; thus, the authors did not evaluate actual colour change of dental tissues, and used a minimal ozone concentration (140 ppm) for four minutes^12^.

In view of to the present outcome, it might be useful to apply ozone after H_2_O_2_ as has been utilised for dental bleaching because this might decrease both retention time and concentration of H_2_O_2_, thus possibly obtaining better bleaching effects. Additionally, this should minimize the chance for tissue irritation and could lead to less post bleaching sensitivity, reduce treatment costs and duration, and enhance patients’ compliance with treatment. The delivered ozone is more controlled by the care provider since the supplying device permits adequate control of delivery site, volume, flow rate and concentration of ozone. Furthermore, the application of ozone does not involve light activation, is quick, less costly, convenient, less irritant to soft tissues, and does not induce tooth sensitivity.

A limitation of the present investigation might be that this research was carried out within clinical settings that are more difficult to monitor if compared to laboratory investigations. Nevertheless, the investigation settings were thoroughly standardized to ensure maximum control of the implemented methodologies and shade assessments. Moreover, the tested sample size was equivalent to or larger than earlier studies in this area^6,9,28^.

Future clinical studies are advocated on larger samples within clinical settings to verify the long-term bleaching outcomes of ozone on natural teeth. Moreover, further research is required to investigate the potentials of ozone for bleaching difficult dental stains like tetracycline or fluorosis staining. Additionally, it would seem appealing to establish the minimum peroxide concentration, which can be applied together with ozone to achieve bleaching outcomes similar to 38% hydrogen peroxide in the same time intervals. Decreasing hydrogen peroxide concentrations to satisfyingly bleach teeth would be advantageous because of the possible clinical benefits by avoiding the side effects of bleaching using higher levels of peroxide^26,27^.

## Conclusion

Within the limitations of the current study, it can be concluded that bleaching efficacy of H_2_O_2_ (20 minutes) will be boosted by a 60-second application of ozone, thus leading to lighter tooth shades, and this is considered irrespective of implementing ozone before or following H_2_O_2_. Using ozone after H_2_O_2_ does not result in increased bleaching sensitivity, while the latter will be observed when applying ozone before H_2_O_2_ or with conventional bleaching alone. Thus, the efficacy of the H_2_O_2_/ozone combination is regarded as advantageous and clinically meaningful when striving for satisfying and rapid bleaching effects. Additional clinical research assessing the acknowledged efficiency of the peroxide/ozone combination is suggested.

### Data availability

The datasets generated and/or analysed during the current study are available from the corresponding author on reasonable request.
